# An experimental study on thermal conductivity and viscosity of nanofluids containing carbon nanotubes

**DOI:** 10.1186/1556-276X-9-151

**Published:** 2014-03-28

**Authors:** Rad Sadri, Goodarz Ahmadi, Hussein Togun, Mahidzal Dahari, Salim Newaz Kazi, Emad Sadeghinezhad, Nashrul Zubir

**Affiliations:** 1 Department of Mechanical Engineering, Faculty of Engineering, University of Malaya, Kuala Lumpur 50603, Malaysia; 2 Department of Mechanical and Aeronautical Engineering, Clarkson University, Potsdam, NY 13699, USA

**Keywords:** Multi-walled carbon nanotubes, MWCNTs, Nanofluids, Thermal conductivity, Viscosity, Dispersant, Surfactant, Gum arabic, SDBS, SDS

## Abstract

Recently, there has been considerable interest in the use of nanofluids for enhancing thermal performance. It has been shown that carbon nanotubes (CNTs) are capable of enhancing the thermal performance of conventional working liquids. Although much work has been devoted on the impact of CNT concentrations on the thermo-physical properties of nanofluids, the effects of preparation methods on the stability, thermal conductivity and viscosity of CNT suspensions are not well understood. This study is focused on providing experimental data on the effects of ultrasonication, temperature and surfactant on the thermo-physical properties of multi-walled carbon nanotube (MWCNT) nanofluids. Three types of surfactants were used in the experiments, namely, gum arabic (GA), sodium dodecylbenzene sulfonate (SDBS) and sodium dodecyl sulfate (SDS). The thermal conductivity and viscosity of the nanofluid suspensions were measured at various temperatures. The results showed that the use of GA in the nanofluid leads to superior thermal conductivity compared to the use of SDBS and SDS. With distilled water as the base liquid, the samples were prepared with 0.5 wt.% MWCNTs and 0.25% GA and sonicated at various times. The results showed that the sonication time influences the thermal conductivity, viscosity and dispersion of nanofluids. The thermal conductivity of nanofluids was typically enhanced with an increase in temperature and sonication time. In the present study, the maximum thermal conductivity enhancement was found to be 22.31% (the ratio of 1.22) at temperature of 45°C and sonication time of 40 min. The viscosity of nanofluids exhibited non-Newtonian shear-thinning behaviour. It was found that the viscosity of MWCNT nanofluids increases to a maximum value at a sonication time of 7 min and subsequently decreases with a further increase in sonication time. The presented data clearly indicated that the viscosity and thermal conductivity of nanofluids are influenced by the sonication time. Image analysis was carried out using TEM in order to observe the dispersion characteristics of all samples. The findings revealed that the CNT agglomerates breakup with increasing sonication time. At high sonication times, all agglomerates disappear and the CNTs are fragmented and their mean length decreases.

## Background

In the recent years, there has been a definite need for energy conservation and thermal management due to the increasing demand for power and the rising energy cost. Heating and cooling processes play the major role in many energy systems; therefore, there is a need to enhance heat transfer and energy efficiency of these thermal management systems. The conventional methods for heat transfer in many systems are using fluids such as water, ethylene glycol and mineral oils. However, the thermal efficiency of these flow systems is hampered by the low heat transfer performance due to the low thermal conductivity of base fluids [1-3]. Nanofluids have been shown to have excellent thermal properties (in particular, thermal conductivity and convective heat transfer coefficient) compared to simple base fluids [2,4-13].

Much fundamental research over the past decade has shown that CNTs have a thermal conductivity that is an order of magnitude higher than copper. The thermal conductivity was found to be approximately 3,000 W/m.K and ~6,000 W/m.K, respectively, for multi-walled carbon nanotubes (MWCNTs) and single-walled carbon nanotubes (SWCNTs) [14,15], which indicates that CNTs have the potential to improve the thermal conductivity of base fluids including water, ethylene glycol and mineral oils.

Developing and effective dispersion of MWCNTs is one of the critical steps involved in the preparation of CNT nanofluids from the base liquids. Dispersing MWCNTs in base fluids is a challenging task due to the high aspect ratio of the nanotubes and strong van der Waals forces between the carbon surfaces as well as the hydrophobic nature of MWCNTs. In addition, MWCNTs tend to entangle among themselves and form clusters and agglomerates when they are dispersed. Various dispersants have been used in previous studies to stabilize CNTs such as sodium dodecylbenzene sulfonate (SDBS) [16], sodium dodecyl sulfate (SDS) [4], Nanosperse AQ (NanoLab Inc., Waltham, MA, USA) [17], hexadecyltrimethyl ammonium bromide (CTAB) [17], chitosan [18] and gum arabic (GA) [6,7]. Cationic dispersants such as hexadecyltrimethyl ammonium bromide, Gemini-type [19] and mixed cationic-anionic [20] were shown to be effective in stabilizing CNTs and various metal particles [21] at low concentrations. High concentrations of cationic Gemini dispersant results in increased multi-walled nanotube (MWNT) sediment, which decreases thermal conductivity enhancement of MWNT nanofluids [22]. Chitosan was also found to be an effective stabilizer for dispersing CNTs in water [18], and it possesses the advantage that it is biocompatible and is a natural polymer isolable from crustacean cells [23]. The addition of SDS improves the stability of CNT nanoparticles in aqueous suspensions. Wusiman et al. [24] showed that CNT nanofluids with SDBS have higher thermal conductivity compared to those with SDS dispersants. Bystrzejewski et al. [25] studied MWCNT suspensions in which SDS and SDBS were used as anionic dispersants and it was found that both dispersants formed stable CNT suspensions. The SDBS dispersant has a higher dispersion power than SDS, by 26% to 45%. They also found that the dispersant's structure influences the diameter distribution of CNTs. The CNTs suspended in SDBS solutions show an increased similarity to narrower CNTs, whereas nanotubes suspended in SDS solutions give the same diameter distribution as pristine CNTs. However, experiments on SDBS solutions above 60°C to 70°C showed that the dispersant results in destabilization of nanofluids [16]. Some researchers used a novel method to disperse nanotubes [6,26], which involves exploiting the physical adsorption characteristics of GA, which is a natural polysaccharide produced by *Acacia senegal* trees. GA was shown to assist the dispersion of CNTs and thus this method can be used for both SWCNTs and MWCNTs. According to previous studies, GA is suitable for dispersing CNTs. However, GA results in an increased viscosity when it is added in small quantities to base fluids such as distilled water. High viscosity is undesirable in nanofluids due to the fact that the nanotubes will stick to the walls of the sample bottles as well as on surfaces of the measuring instruments. A highly viscous nanofluid will also increase the pumping power for commercial applications. Hence, it is crucial that dispersants are added in optimum quantities [6]. Previous studies have shown that 0.25 wt.% GA is a suitable quantity to disperse MWCNTs.

A significant thermal conductivity enhancement was observed by Choi et al. [27] for MWCNTs dispersed in synthetic poly (α-olefin) oil. They reported a nonlinear thermal conductivity enhancement of up to 160% for only 1 vol.% of CNTs. The unusual thermal conductivity enhancement which was significantly higher than the theoretical prediction for nanofluids was attributed to the high thermal conductivity of particles (3,000 W/m.K), as well as the size and shape of the nanotubes. Xie et al. [28] presented the thermal conductivity enhancement for base fluids such as water, ethylene glycol and decene, while Assael et al. [4,17] investigated aqueous MWCNT nanofluids with SDS, CTAB and Nanosperse AQ dispersants. However, both studies showed that the thermal conductivity enhancement for the solutions was less than that obtained by Choi et al. [27]. Xie et al. [28] obtained a maximum thermal conductivity enhancement of only 2% for 1 vol.% nanotubes in decene, while Assael et al. [4] obtained an enhancement of 38% for 0.6 vol.% MWCNTs-water nanofluid with 0.1 wt.% SDS. In 2004, Wen and Ding [16] observed a thermal conductivity enhancement of 23% and 28% for 0.84 vol.% MWCNT-water nanofluids with SDBS at 20°C and 40°C, respectively. Their results were comparable to the findings of Xie et al. [28] and Assael et al. [4]. However, Wen and Ding [16] stated that the discrepancies between their findings with those of Choi et al. [27] were due to differences in interfacial resistance and thermal conductivity of carbon nanotubes used in their study. In addition, Choi et al. [27] used poly-α olefin as the base fluid which has a lower thermal conductivity than water. Hence, even though the percentage thermal conductivity enhancement was high, the absolute thermal conductivity enhancement was not as high as expected. SDBS also fails at elevated temperatures [16]. In 2006, Ding et al. [6] published a set of thermal conductivity data in which GA was used as the dispersant. They attained a thermal conductivity enhancement of 28% and 79% at 25°C and 30°C, respectively, for 1 wt.% of MWCNTs in water. The thermal conductivity measurements revealed that the effective thermal conductivity increases with an increase in temperature and CNT concentration; however, the dependence of conductivity on temperature was much more significant. The thermal conductivity enhancement was slightly higher than those reported by Assael et al. [4], Xie et al. [28] and Wen and Ding [16], which was attributed to the thermal properties and aspect ratio of the CNTs used, as well as liquid-CNT interfacial resistance. Furthermore, the base liquid also plays a role for the discrepancies in their findings.

Recently, Chen and Xie [22] used cationic Gemini and achieved a thermal conductivity enhancement of 34.3% and 5.6% at 65°C and 5°C, respectively, with a volume fraction of 0.6%. They showed that temperature has a significant effect on the thermal conductivity enhancement of MWCNT-water nanofluids stabilized by cationic Gemini dispersant. The enhancement was slightly less than the value reported by Ding et al. [6]. Indhuja et al. [13] reported a thermal conductivity enhancement of 8% and 33% at of 28°C and 60°C, respectively, for a concentration of 5 wt.% MWCNTs.

In 2004, Jang and Choi [29] postulated another theory in which the Brownian motion of nanoparticles is the potential factor for increasing the thermal conductivity of nanofluids at elevated temperatures. They proposed that the viscosity of base fluids decreases with increasing temperature, which increases the Brownian motion of nanoparticles. It was postulated that the convection-like effects were induced by Brownian motion, which consequently increases the thermal conductivity. However, Keblinski et al. [30] showed that the Brownian motion unlikely influences the thermal conductivity of nanofluids. Amrollai et al. [31] explored the effects of ultrasonication time on the thermal conductivity and sedimentation of carbon nanotube-ethylene glycol nanofluids and discovered that the effective thermal conductivity is strongly dependent on the temperature and volume fraction of CNT nanofluids. The thermal conductivity increases with an increase in ultrasonication time, which may be attributed to Brownian motion and inter-particle potential. Yang et al. [32] investigated the impact of sonication energy/time on the thermal conductivity of nanotube-oil suspensions and observed a decrease in thermal conductivity with an increase in sonication energy/time. Garg et al. [7] investigated the effects of ultrasonication energy on the thermal conductivity and viscosity of MWCNTs-water nanofluids dispersed using GA. It was found that an optimum ultrasonication time is sufficient to disperse nanotubes without causing breakup. A thermal conductivity enhancement of 20% was achieved for 1 wt.% MWCNT-water nanofluids for optimum ultrasonication energy of 113 J/g (40 min sonication time).

Recently, Ruan and Jacobi [10] showed that the thermal conductivity of MWCNTs-ethylene glycol nanofluids increases nonlinearly with an increase in sonication time/energy and achieved a maximum thermal conductivity enhancement of 23% at a concentration of 0.5 wt.% with an elapsed sonication time of 1,355 min. They observed that the sonication process reduces the size of the agglomerates as well as the length of CNTs. However, the reduction in agglomerate size is found to be more significant compared to the reduction in length. It can be observed that ultrasonication is the conventional method to break up agglomerates and promote dispersion of nanoparticles in base fluids. However, there are limited research reports regarding the effects of sonication on CNT nanofluid properties in the open literature.

A comparison of the thermal conductivity of various CNT nanofluids reported in the literature is presented in Table 1. The particle volume concentration, particle size, thermal conductivity, base liquid and dispersant used are also listed in this table.

**Table 1:** Summary of experimental investigations on thermal conductivity of CNT nanofluids

**Researcher/year/reference**	**Particle**	**Base fluid**	**Average particle size**	**Concentration (vol.%/wt.%)**	**Thermal conductivity enhancement/ratio**	**Note**
Choi et al. 2001 [27]	MWCNT	Oil	25 nm × 50 μm	1 vol.%	150%	-
Xie et al. 2003 [28]	MWCNT	Decene/ethylene glycol/water	15 nm × 30 μm	1 vol.%	20%/13%/7%	-
					(1.20, 1.13, 1.07)	
Assael et al. 2004 [4]	MWCNT	(+ SDS)-water	100 nm × 70 μm	0.6 vol.%	38%	-
Wen and Ding 2004 [16]	MWCNT	(+ Sodium dodecyl benzene)-water	20-60 (diameter)	0.04-0.84 vol.%	1.04-1.24	Temperature effect (20°C)
Wen and Ding 2004 [16]	MWCNT	(+ Sodium dodecyl benzene)-water	20-60 (diameter)	0.04-0.84 vol.%	1.05-1.31	Temperature effect (45°C)
Assael et al. 2005 [17]	MWCNT	(+ CTAB)-water	L 10 μm	0.6 vol.%	1.34	-
			OD 100-250		34%	
Assael et al. 2005 [17]	DWCNT	(+ CTAB)-water	5 nm (diameter)	1.00 vol.%	1.08	Dispersant effect
Assael et al. 2005 [17]	DWCNT	(+ CTAB)-water	5 nm (diameter)	0.75 vol.%	1.03	-
Liu et al. 2005 [33]	MWCNT	Ethylene glycol	20-50 (diameter)	0.20-1.00	1.02-1.12	Two-step method
				(1 vol.%)	(12.4%)	
Liu et al. 2005 [33]	MWCNT	(+ *N*-hydroxysuccinimide)-engine oil	20-50 (diameter)	1.00-2.00 (2 vol.%)	1.09-1.30 (30%)	Two-step method
Marquis and Chibante 2005 [34]	SWCNT	(+ Dispersant)-diesel oil (Shell/Rotella 15 W-40)	(10-50) × (0.3-10 μm)	0.25-1.00	1.10-1.46	Two-step method
Ding et al. 2006 [6]	MWCNT	(+ Gum arabic)-water	-	0.05-0.49	1.00-1.10	Temperature effect (20°C)
Ding et al. 2006 [6]	MWCNT	(+ Gum arabic)-water	-	0.05-0.49	1.07-1.27	Temperature effect (25°C)
Ding et al. 2006 [6]	MWCNT	(+ Gum arabic)-water	-	0.05-0.49	1.18-1.8	Temperature effect (30°C)
Hwang et al. 2006 [23]	MWCNT	Mineral oil	-	0.5	1.09	-
Yang et al. 2006 [32]	MWCNT	(+ Polyisobutene succinimide)-poly(α-olefin	-	0.04-0.34	1.06-3.00	Two-step method
Amrollahi et al. 2008 [31]	MWCNT	Ethylene glycol	OD 1-4	2.5 vol.%	20%	Temperature effect (25°C-50°C)
			ID 0.8-1.1			
Amrollahi et al. 2008 [31]	MWCNT	Ethylene glycol	OD 1-4	0.5 vol.%	1.05-1.2	Ultrasonication effect (1-24 h)
			ID 0.8-1.1	2.5 vol.%	1.1-1.32	
Garg et al. 2009 [7]	MWCNT	(+ Gum arabic)-water (35 °C)	OD 10-20 nm	1 wt.%	20%	Ultrasonication effect (40 min)
			L 0.5-40 μm			
Chen and Xie 2010 [22]	MWCNT	(+ Cationic Gemini)-water	OD 30-50 nm	0.6 vol.%	5.6%-34%	Temperature effect (5°C-65°C)
			L ~ 20 μm			
Phuoc et al. 2011 [18]	MWCNT	(+ Chitosan)-water	OD 20-30 nm	(0.5-3) wt.% (0.24-1.43) vol.%	2.3%-13%	Two-step method (35°C)
			ID 5-10 nm			
			L 10-30 μm			
Singh et al. 2012 [35]	MWCNT	Ethylene glycol + water	D 60-30 nm	0.4 wt.%	72%	Nitric and sulfuric acid treatment
			L 5-15 μm			
Kumaresan and Velraj 2012 [36]	MWCNT	(+ SDBS)-ethylene glycol + water	D 30-50 nm	0.45 vol.%	19.75%	Temperature effect (40°C)
			L 10-20 μm			
Ruan and Jacobi 2012 [10]	MWCNT	Ethylene glycol	OD 10-30	0.5 wt.%	23%	Ultrasonication effect 1,355 min
			L 10-30			
			ID 5-10			
Indhuja et al. 2013 [13]	MWCNT	(+ Gum arabic)-water	ID 10 nm	(0.14-0.24) vol.%	0.61-0.67 (3.2%-10%)	Effect of concentration
			L 5-15 μm			
Indhuja et al. 2013 [13]	MWCNT	(+ Gum arabic)-water	ID 10 nm	0.5 wt.%	0.66-0.93 (8%-33%)	Temperature effect (28°C-60°C)
			L 5-15 μm			
Indhuja et al. 2013 [13]	MWCNT	(+ Gum arabic)-water	ID 10 nm	0.3 wt.%	0.63-0.88 (5%-26%)	Temperature effect (28°C-60°C)
			L 5-15 μm			

DWCNT, double-walled carbon nanotubes; ID, inside diameter; OD, outside diameter; L, length (μm); D, density.

In this study, the effects of ultrasonication time and type of dispersants used on the thermal properties of MWCNT nanofluids were examined. Three types of dispersants were used in the experiments, namely, GA, SDBS and SDS, and the most superior dispersant was selected for further experiments on MWCNT nanofluid suspensions. The thermal conductivity and viscosity of the nanofluid suspensions were measured at various temperature settings and different sonication times. It was shown that the dispersant used, temperature, sonication time and shear rate significantly affect the thermo-physical properties of MWCNT nanofluids.

## Methods

Distilled water, GA, SDBS, SDS and MWCNTs were used in preparing the aqueous suspensions. The nanotubes were sourced from Nanostructured & Amorphous Materials Inc., Houston, TX, USA. The properties of the MWCNTs are shown in Table 2.

**Table 2:** Properties of multi-walled carbon nanotubes

	**Outside diameter, OD (nm)**	**Length, *L *** **(um)**	**Density, μ (g/cm**^**3**^**)**	**Purity (%)**	**Thermal conductivity, k (W/m.K) at 300 K**	**Specific surface area, SSA (m**^**2**^**/g)**
Sample (MWCNT)	20-30	10-30	~2.1	>95	2,800	110-130

**Figure 1:**
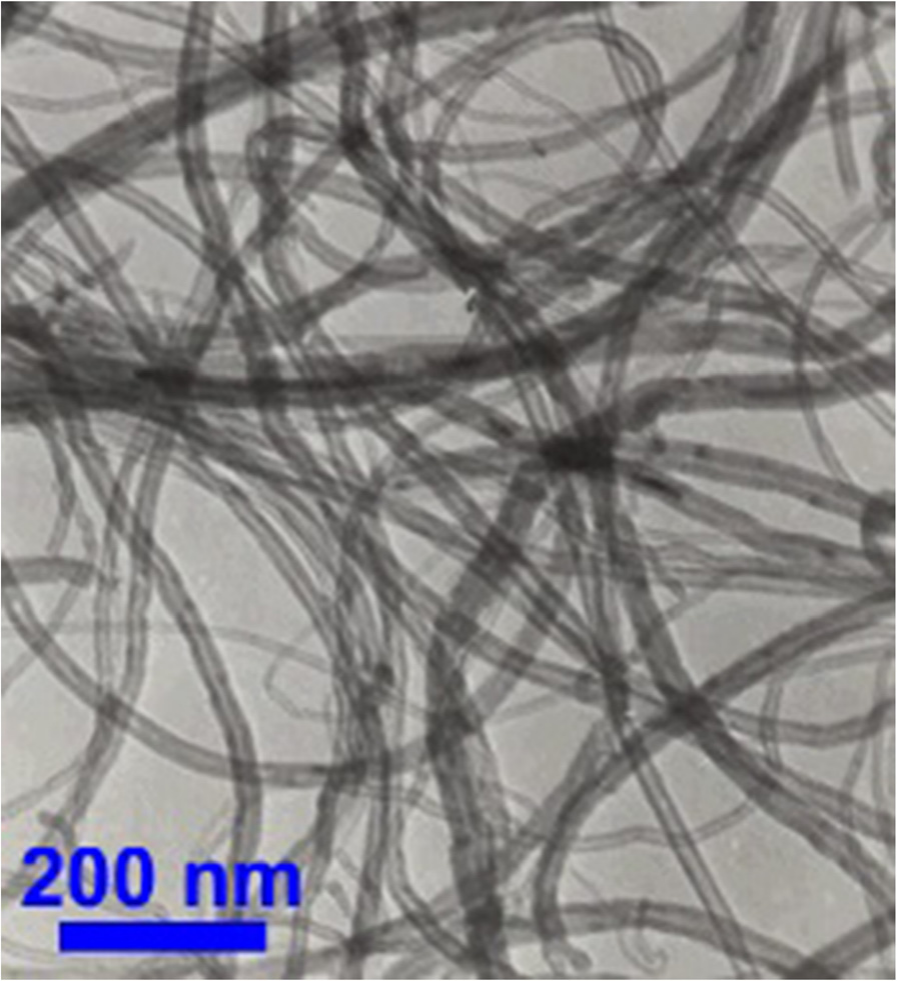
TEM image of multi-walled carbon nanotubes (as received).

The nanotubes were produced by chemical vapour deposition (CVD) process. Figure 1 represents a transmission electron microscopy (TEM) image of the MWCNTs as received. It is seen that the nanotubes are not only entangled but also form agglomerates in the absence treatment. Gum arabic, sodium dodecylbenzene sulfonate, and sodium dodecyl sulfate were purchased from Sigma-Aldrich Co., Selangor, Malaysia.

### Preparation of MWCNT-water nanofluids

**Figure 2:**
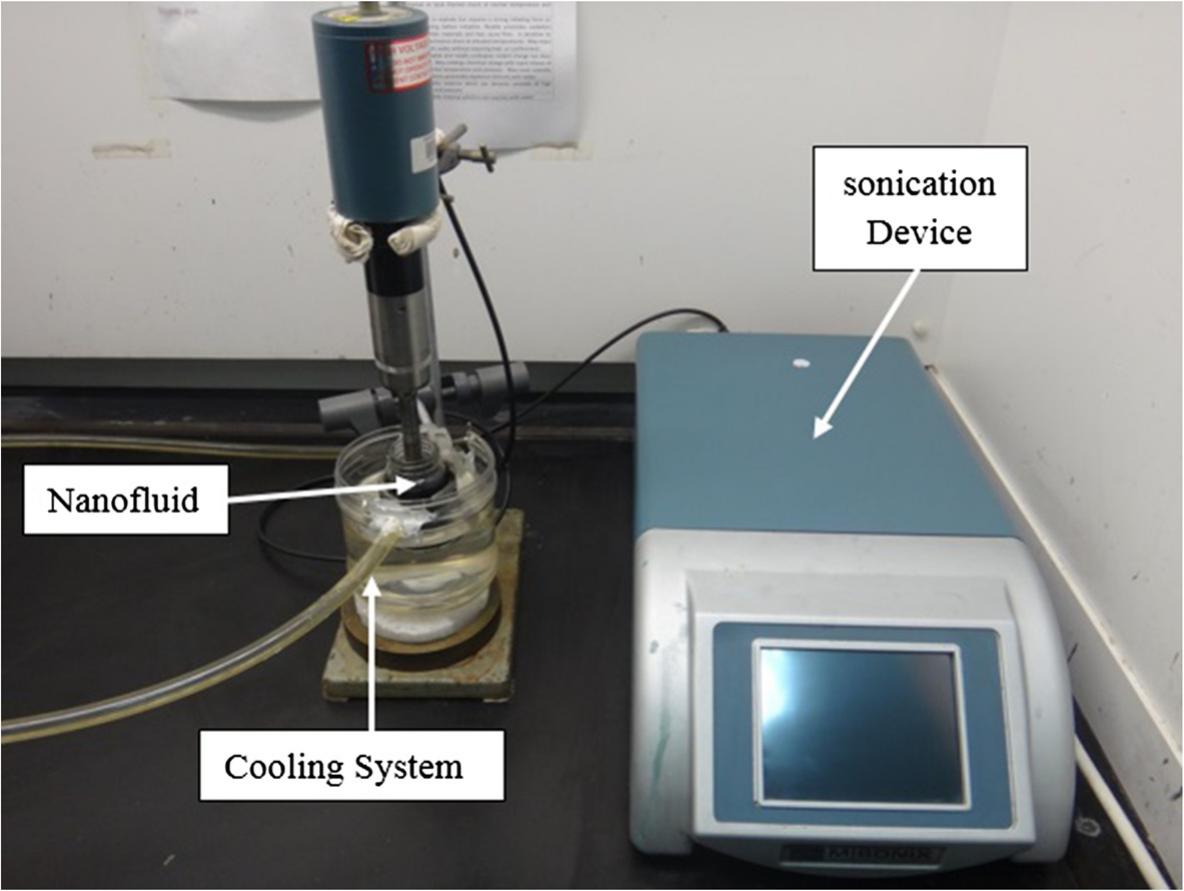
Nanofluids preparation set-up.

Since the surface of MWCNTs is hydrophobic and water is a polar liquid, GA, SDBS and SDS were used to disperse CNTs in distilled water. The required amount of base fluid was first poured into 60-ml glass beakers. Following this, 0.25 and 0.5 wt.% SDBS and SDS, respectively, were added into the base fluid. Since GA concentration of 0.25 wt.% was used in the literature and was proven to be the optimum amount for preparing water-MWCNTs nanofluids [6], the same concentration was also used in this study, 0.25 wt.%, and the suspensions were dispersed using a magnetic stirrer. Typically, 0.5 wt.% of MWCNTs was added into the solutions once the dispersants were dissolved completely and homogeneous solutions were obtained. Each solution was then ultrasonicated for 20 min using an ultrasonication probe until a homogeneous suspension was achieved. During the sonication, it was observed that bubbles formed and collapsed. It is conjectured that the resulting shock from this cavitation process (collapsing bubbles) breaks up the nanotube agglomerates. However, sonication also generates heat which results in an increase in the nanofluid temperature. To avoid the variation of the temperature, a cooling system was employed to maintain the sample temperature at about 25°C. Ultrasonic treatment was performed using an ultrasonic liquid processor (Misonix Inc., Farmingdale, New York, NY, USA) having an output of 600 W and fitted with 20-kHz converter. The most effective dispersant was selected by examining the thermal performance of nanofluids dispersed with different dispersants. Following this, CNT nanofluids were prepared using 0.5 wt.% MWCNTs mixed with the most effective dispersant identified from the previous step. The solutions were then sonicated at different ulrasonication times (2, 7, 10, 20, 30 and 40 min). The sample preparation set-up is shown in Figure 2.

### Thermal conductivity measurement procedure

**Figure 3:**
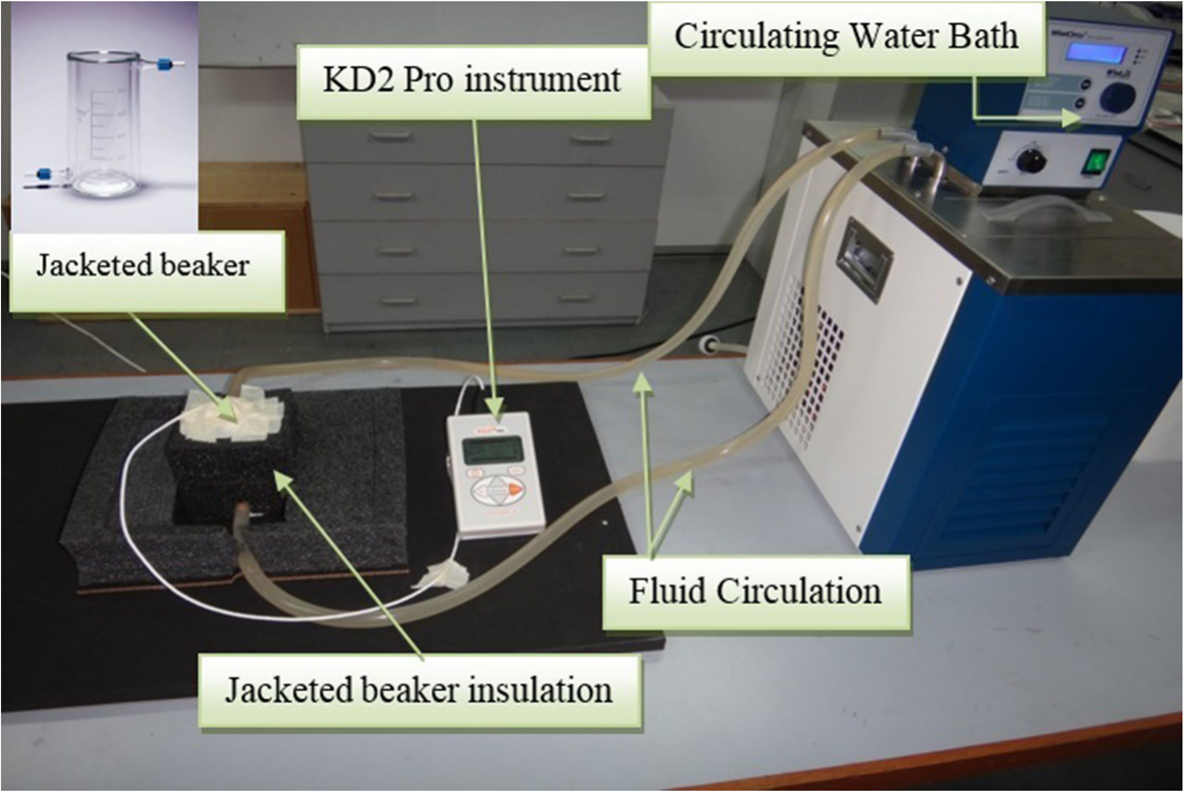
Experimental set-up for thermal conductivity measurements.

The thermal conductivity of nanofluids was measured using KD2 Pro instrument (Decagon, Pullman, WA, USA), as shown in Figure 3. The instrument is based on the working principle of a transient hot wire method used in previous works [4,6,17,37] and has an accuracy of about 5%. A single needle sensor (1.3-mm diameter × 60-mm long) was also used for thermal conductivity measurements and was installed in a jacketed beaker connected to a water bath. The experimental set-up ensures temperature stability in order to obtain accurate measurements. The effects of temperature on the thermal conductivity of nanofluids were investigated for all samples. Water bath (WiseCircu, Witeg Labortechnik GmbH, Wertheim, Germany) with 0.1°C accuracy was used to keep the temperature at 20°C, 25°C, 30°C, 35°C, 40°C, 45°C when measuring the thermal conductivity of nanofluid samples. Nanofluids were placed into the jacketed beaker, and the temperature was kept constant for each test. The water bath has an inlet and outlet tube for flowing and circulating water at a specific temperature in order maintain temperature stability. For each sample, measurements were taken at every 30 min, and the thermal conductivity of the sample is evaluated as the mean of ten readings at the same temperature.

### Viscosity measurement procedure

The viscosity of the CNT nanofluids and water was measured using a rotational rheometer (Physica, MCR-301, Anton Paar, Graz, Austria). The instrument consists of two parallel cylindrical surfaces with a gap of 0.500 mm; the mobile cylinder has a diameter of 50 mm. The viscosity of the suspensions was determined by varying the shear rate in the range of 10 to 140/s. Measurements were taken at temperatures of 15°C, 30°C, 45°C and were repeated four times for each experiment to obtain accurate results. The maximum deviation was found to be less than 5%.

### TEM imaging

Images of the nanofluids were obtained using TEM (LIBRA 120; Carl Zeiss, Oberkochen, Germany). A drop of the MWCNT nanofluid solution was placed onto a carbon-coated copper grid and dried at room temperature after removal of excess solution by filter paper.

## Results and discussion

### Thermal conductivity results

#### Base liquid

**Figure 4:**
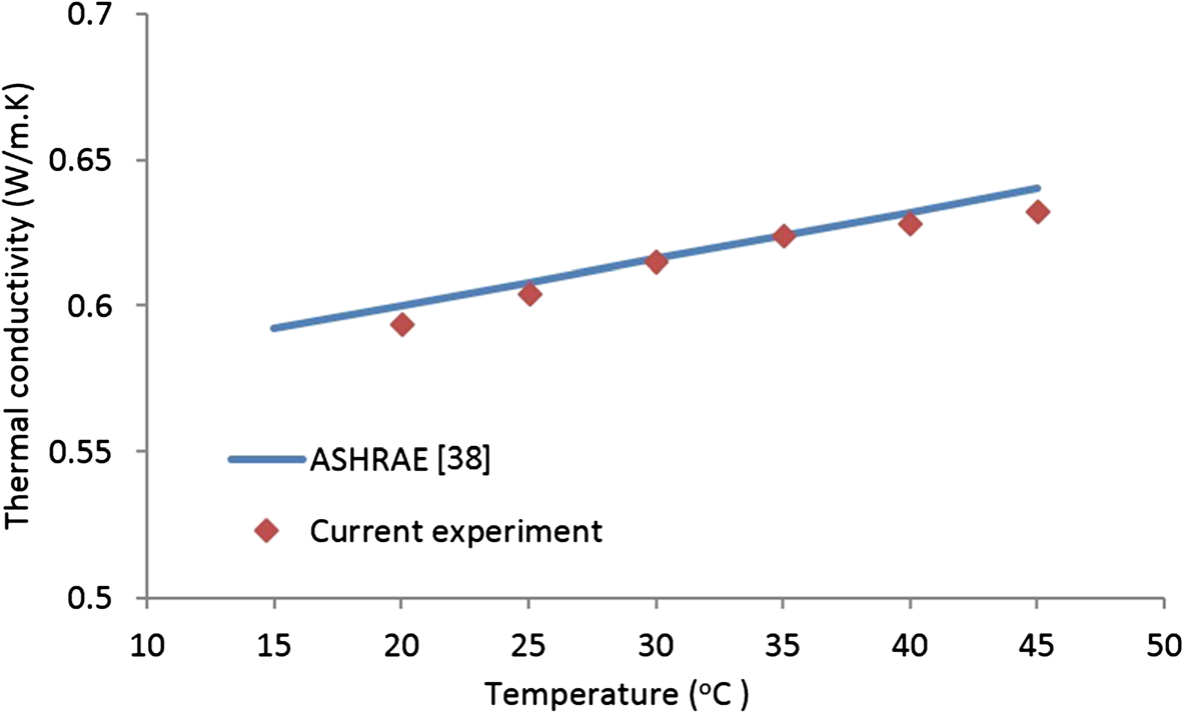
Benchmark test for water thermal conductivity.

The thermal conductivity of the distilled water as a function of temperature was measured, and the results were compared with the American Society of Heating, Refrigerating and Air Conditioning Engineers (ASHRAE) data [38] in order to establish the reliability and accuracy of the measurements. Figure 4 compares the measured water thermal conductivity with the ASHRAE data [38]. The experimental results show good agreement with the reference data, as all measurements are within 1.2% of the ASHRAE values. Thus Figure 4 verifies the reliability of the experimental procedure for measuring thermal conductivity of water-based nanofluids. As is expected, this figure also shows that the thermal conductivity of distilled water increases with the increase of temperature.

#### Effects of SDBS, SDS and GA dispersants on base fluid

**Figure 5:**
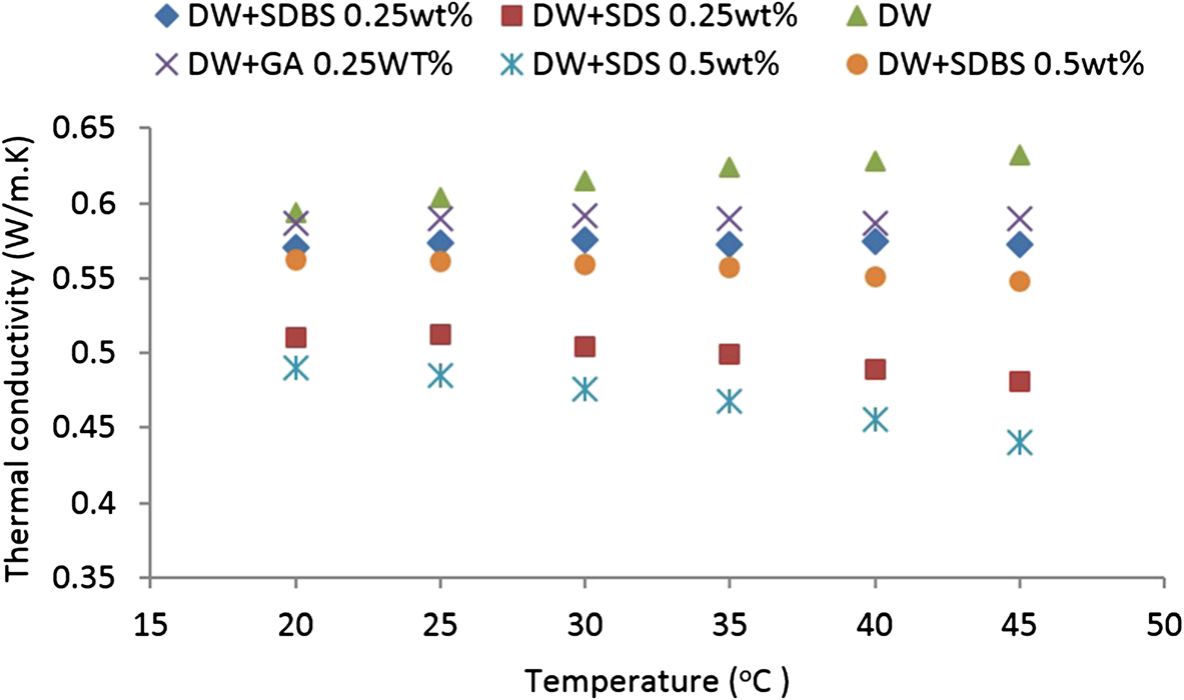
Effects of SDBS, SDS and GA on thermal conductivity of base fluid.

It is deemed necessary to investigate the influence of dispersants on the thermal conductivity of distilled water in order to understand the impact of these dispersants on nanofluids. It is also important to identify which dispersant is the most suitable for generating the aqueous CNT suspensions. In this study, 0.25 wt.% GA, SDBS and SDS, as well as 0.5 wt.% SDBS and SDS were dispersed in distilled water and the corresponding thermal conductivity of these solutions were measured. The resulting conductivities are compared graphically with those for distilled water in Figure 5. This figure shows that the dispersants suppress the thermal conductivity of distilled water. Another notable trend is that the thermal conductivity of water decreases with an increase in dispersant concentrations. More importantly, Figure 5 shows that the increasing trend of distilled water conductivity with temperature changes to a decreasing trend with the addition of dispersants.

Comparison of the three dispersants added to the distilled water at the same concentrations shows that GA leads to the highest thermal conductivity compared to SDS and SDBS, that is, GA has a smaller effect on reducing the thermal conductivity of the base fluid compared to the other two dispersants (SDS and SDBS). The thermal conductivity of the solution containing SDS exhibits a higher decrease of thermal conductivity at higher temperatures compared to SDBS and GA dispersants. It should be pointed out that all samples are sonicated for 20 min before testing.

#### Effects of GA, SDBS and SDS dispersants on carbon nanotube nanofluids

**Figure 6:**
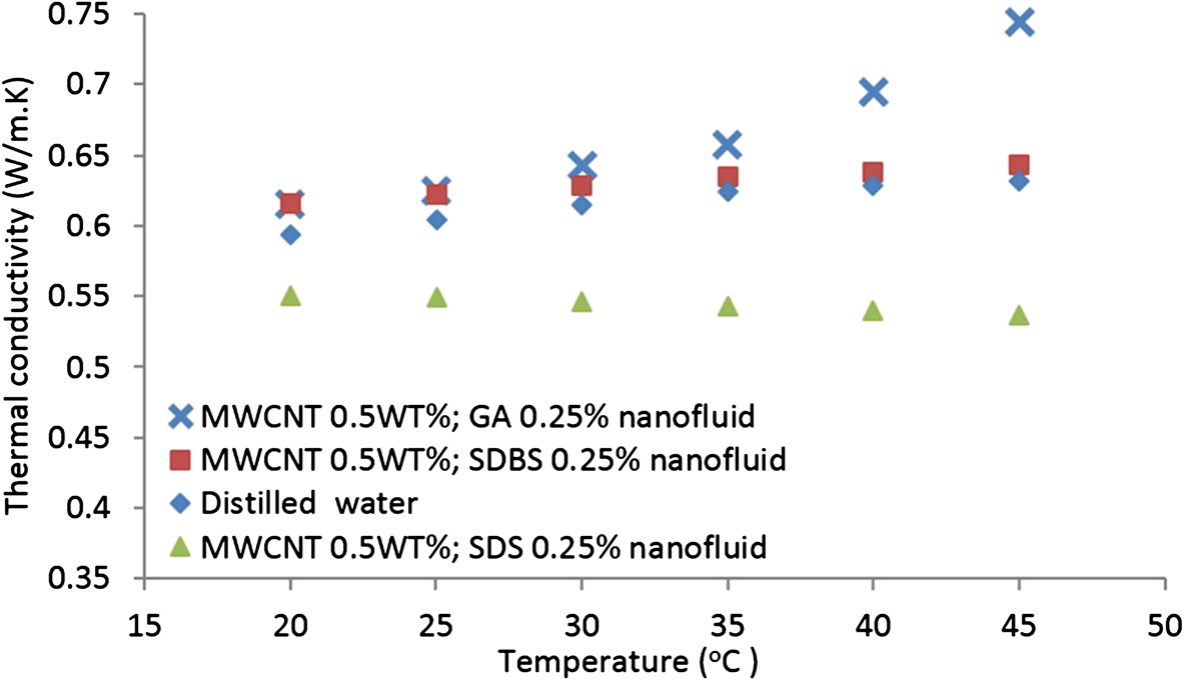
Comparison of thermal conductivity of CNT nanofluids containing GA, SDBS and SDS dispersants.

Figure 6 shows the effects of GA, SDBS and SDS dispersants on the thermal conductivity of CNT nanofluids. Here the concentration for each dispersant is 0.25 wt.% and the amount of MWCNTs is 0.5 wt.%, and all samples are sonicated for 20 min. This figure shows that the thermal conductivity of nanofluids containing GA dispersant is larger than the distilled water and also substantial increases with increasing temperature. Figure 6, however, shows that the thermal conductivity of CNT nanofluid with SDS dispersant is lower than the distilled water and decreases slightly with increasing temperature. This is due to the fact that the SDS dispersant significantly suppresses the thermal conductivity of the base fluid, as was seen in Figure 5. The thermal conductivity of nanofluids containing SDBS dispersant is slightly more than the distilled water and increases mildly with increasing temperature. From Figures 5 and 6, it is evident that GA dispersant is superior to both SDS and SDBS in regard to enhancing the thermal performance of CNT suspensions. Hence, GA is the recommend choice for dispersing MWCNTs. In the subsequent section, unless stated otherwise, GA dispersant was used.

#### Effects of ultrasonication time and temperature

**Figure 7:**
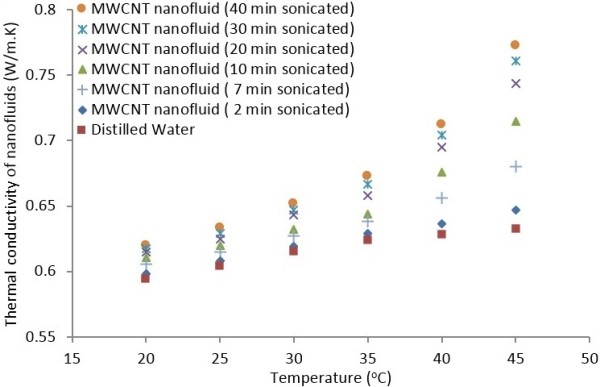
Effects of ultrasonication time and temperature on thermal conductivity of nanofluids.

Figure 7 shows the effects of ultrasonication time and temperature on the thermal conductivity of MWCNT nanofluids containing 0.5 wt.% MWCNTs. The temperature was varied from 20°C to 45°C, and the thermal conductivity data were recorded using KD2 Pro Thermal Properties analyzer. The measurements were taken for various sonication times and temperatures. The data shown in Figure 7 are the mean of ten readings, and the accuracy of the measuring instrument is 5%.

Figure 7 again confirms that the thermal conductivity of MWCNT nanofluids increases with an increase in temperature. The thermal conductivity data for distilled water are also included in this figure for comparison. Clearly, the thermal conductivity of nanofluids is markedly larger than that for distilled water especially at temperatures higher than 35°C. Unlike water, the thermal conductivity of nanofluids first increases slightly with temperature and then increases sharply with temperature after 30°C, that is, the increase in the thermal conductivity at higher temperatures is not solely due to the corresponding increase in thermal conductivity of the base fluid. One possible explanation for this trend is the increased Brownian motions of nanoparticles with temperature. According to Amrollahi et al. [31], Li and Ahmadi [39] and Shams et al. [40], the random Brownian motion of the suspended nanoparticles shows a strong dependence on temperature. The nanoparticle random motions disturb the flow and enhance local mixing. Thus, it is expected that the thermal conductivity of nanofluids will increase sharply with the increase in the suspension temperature. Jang and Choi [29] proposed that the viscosity of the nanofluids decreases with increasing temperature, which also increases the Brownian motion of the nanoparticles. As noted before, Brownian motions set off convection-like effects by dragging the fluid, which enhance thermal conductivity. Ding et al. [6] also observed a strong correlation between temperature and thermal conductivity of MWCNTs dispersed in water.

**Figure 8:**
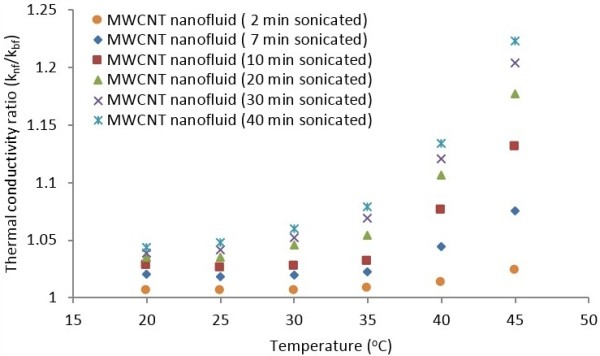
Variation of thermal conductivity ratio as function of temperature at various ultrasonication times.

The ratios of the measured nanofluid thermal conductivity to that of distilled water are evaluated and the results are presented in Figure 8. The variations of thermal conductivity enhancement at various ultrasonication times are tabulated in Table 3. It is seen from the results that the thermal conductivity ratio and thermal conductivity enhancement increases for all MWCNT suspensions, respectively, from 1.2 to 1.22 and 1.01% to 22.31% for the range of temperature and ultrasonication studied. It should be noted that the MWCNT suspensions containing 0.5 wt.% MWCNTs were sonicated from 2 to 40 min. It is observed that the highest thermal conductivity enhancement of 22.31% is achieved for sample 6 (0.5 wt.% MWCNTs, 0.25 wt.% GA, sonication time 40 min) at 45°C.

**Table 3:** Variation of thermal conductivity enhancement (%) as function of temperature at various ultrasonication times

**Concentration (wt.%)**	**Temperature (°C)**	**Sonication times**^**a **^**(min)**					
		**2**	**7**	**10**	**20**	**30**	**40**
0.5	20	0.67	2.02	2.86	3.54	3.87	4.38
	25	0.66	1.82	2.65	3.48	4.14	4.80
	30	0.65	1.95	2.76	4.55	5.20	6.02
	35	0.80	2.24	3.21	5.45	6.89	7.85
	40	1.27	4.46	7.64	10.67	12.10	13.38
	45	2.37	7.59	13.13	17.72	20.41	22.31

^a^Values are expressed in percentage (%).

**Figure 9:**
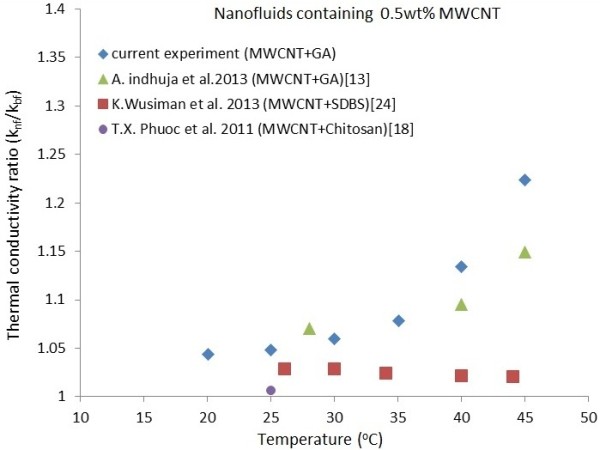
Comparison of thermal conductivity between current experimental data and previous studies for 0.5 wt.% MWCNT nanofluids.

Since the MWCNTs provided by the supplier has an average density of 2.1 g/cm^3^, the volume fraction of the nanopowders used in the experiments is approximately 0.24% by volume. A comparison between the results obtained in this study and those of previous studies is presented in Figure 9. It can be observed that the thermal conductivity ratio in this study is approximately 6% higher than that reported by Indhuja et al. [13] for the 0.5 wt.% MWCNT suspensions dispersed by GA. It is conjectured that this difference is due to the different ultrasonication processor and dispersion method used in these experiments. Phuoc et al. [18] reported a thermal conductivity enhancement of 2.4% and 4.3% for 0.5 and 1 wt.% MWCNT nanofluid (aspect ratio of approximately 500 to 1,000), respectively, at 25°C. Chen and Xie [22] obtained a thermal conductivity enhancement of approximately 15% for the 0.3 vol.% (approximately 0.63 wt.%) MWCNT nanofluid (aspect ratio of approximately 400 to 670) at 45°C. It should be noted that both Phuoc et al. [18] and Chen and Xie [22] used higher CNT concentrations than that used in the present study. Nonetheless, there is a pronounced increase in the thermal conductivity enhancement values achieved in the present work compared to the previous studies. For example, Wusiman et al. [24] reported a thermal conductivity enhancement for 0.5 wt.% MWCNT nanofluid at 40°C that was 10% less than that found in the present study. One reason for the higher values obtained in this study is the type of dispersant (GA) used in the experiments. Phuoc et al. [18], Chen and Xie [22] and Wusiman et al. [24], respectively, used chitosan, cationic Gemini and SDBS dispersants in their studies.

Ding et al. [6] reported a value of 1.1 for the thermal conductivity ratio of 0.5 wt.% MWCNT nanofluid at 20°C, which is much higher than the present experimental data, as well as data of other recent measurements [18,22,24]. The reason for this difference is not known, but it may be associated with the thermo-physical properties of the MWCNTs as well as the dispersion method used. Furthermore, Ding et al. [6] did not provide the information on the aspect ratio (*L*/*D*) of their MWCNTs, which may differ from the aspect ratio of the MWCNTs used in this study (approximately 500 to 1,000).

**Figure 10:**
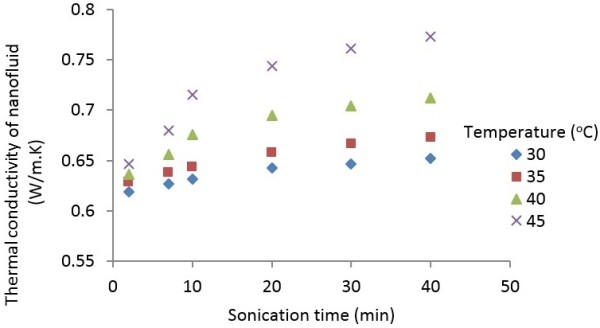
Variation of thermal conductivity as function of ultrasonication time at various temperatures.

Figure 10 shows the thermal conductivity of MWCNT suspensions containing 0.5 wt.% MWCNT nanoparticles as a function of ultrasonication time at various temperatures. It is apparent from Figure 10 that the thermal conductivity of nanofluids initially increases with increasing ultrasonication time, and the rate of increase decreases as sonication time increases or temperature decreases. The effect of ultrasonication is attributed to the breakup of nanoparticle aggregates into smaller clusters. Amrollahi et al. [31] observed that shorter clusters move faster, and there is a higher energy transport within the nanofluid. This observation is consistent with the inverse dependence of Brownian motion with cluster size. Therefore, it is quite likely that increased ultrasonication time leads to a more uniform dispersion of small clusters of MWCNT nanoparticles, which contributes to the increased enhancement of thermal conductivity, as shown in Figure 10. This effect can be observed from TEM images, which are discussed in Section ‘Morphology’.

Assael et al. [4] showed that a decrease in aspect ratio reduces thermal conductivity enhancement. However, Ruan and Jacobi [10] found that a decrease in aspect ratio has negligible effects on thermal conductivity compared to the reduction in cluster size. Amrollahi et al. [31] concluded that the agglomeration of nanoparticles reduces the effective surface area to volume ratio, which reduces the effective area of thermal interaction between the particles and the fluid. This results in a decrease in nanofluid thermal conductivity.

#### Reproducibility of thermal conductivity data for MWCNT nanofluids

**Figure 11:**
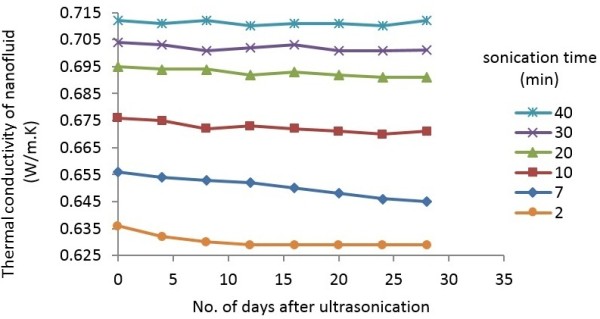
Reproducibility of thermal conductivity data of MWCNT nanofluids at 40°C over 28 days.

One procedure used to examine the stability of nanofluids data and possible signs of visible sedimentation is to experiment with the colloidal suspension properties over a period of time. The thermal conductivity of MWCNT-water nanofluids sonicated for 2 to 40 min was measured for 28 days and the results are plotted in Figure 11. It is seen that the thermal conductivity of nanofluids that are sonicated for short times (2 and 7 min) degrades with the times. However, this figure shows that the deviations in the measured thermal conductivity of nanofluids that are sonicated for 10 to 40 min are negligible. Hence, the thermal conductivity data of the nanofluid suspensions that are sonicated for 10 to 40 min are stable and reproducible for at least over 28 days.

### Viscosity results

**Figure 12:**
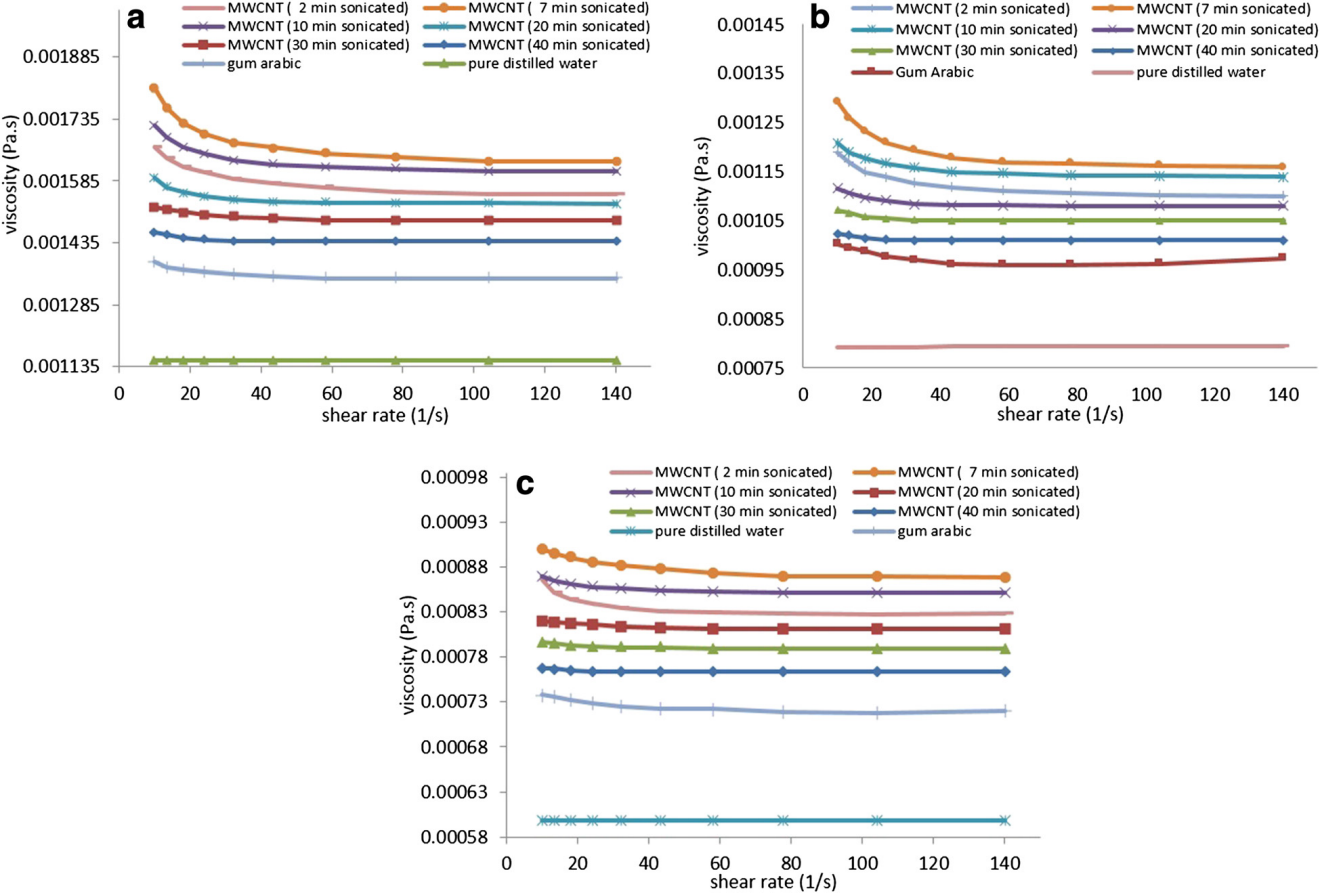
**Variation of dynamic viscosity as function of shear rate at various sonication times. (a)** 15°C. **(b)** 30°C. **(c)** 45°C.

The viscosity of MWCNT-water nanofluids stabilized by GA dispersant was measured as a function of shear rate for various ultrasonication times. Figure 12a,b,c shows, respectively, the results for the nanofluids containing 0.5 wt.% MWCNTs and 0.25 wt.% GA at 15°C, 30°C, and 45°C. The viscosity of the pure distilled water viscosity was also measured prior to measurement, and the results are compared with those from the literature in order to verify the accuracy of the measurement system. The data for the distilled water shows that there are no variations in the dynamic viscosity with increasing shear rate. Unlike water, it is apparent from the results that the MWCNT-water nanofluids behave as a non-Newtonian fluid since the dynamic viscosity varies accordingly with an increase in shear rate. A shear-thinning trend of CNT nanofluids was also observed by Yang et al. [32], Garg et al. [7], Singh et al. [35], Phuoc et al. [18] and Ruan and Jacobi [10], which exhibits due to a decrease in the dynamic viscosity with increasing shear rate. Figure 12 shows that there is a sharp decrease in the viscosity of nanofluids with increase of shear rate at lower shear rates, and the viscosity becomes gradually constant at higher shear rates. The possible reasons are that the nanofluid at the parallel plate is under pressure at high shear rate, which breaks up the CNT clusters and agglomerates. In addition, it is known that elongated fibres align themselves with direction of flow in shear flows [41,42]. Similarly, it is expected that the CNTs also tend to align themselves in the shearing flow direction, which decreases the resistance to the flow and could reduce effective viscosity of nanofluids. Figure 12a,b,c also shows that the viscosity of the MWCNT nanofluids decreases as temperature increases. The increase in sonication time reduces the sharp variation of viscosity with shear rate. The viscosity of MWCNT nanofluids sonicated for 40 min varies smoothly with shear rate and approaches its asymptotic values very quickly.

**Figure 13:**
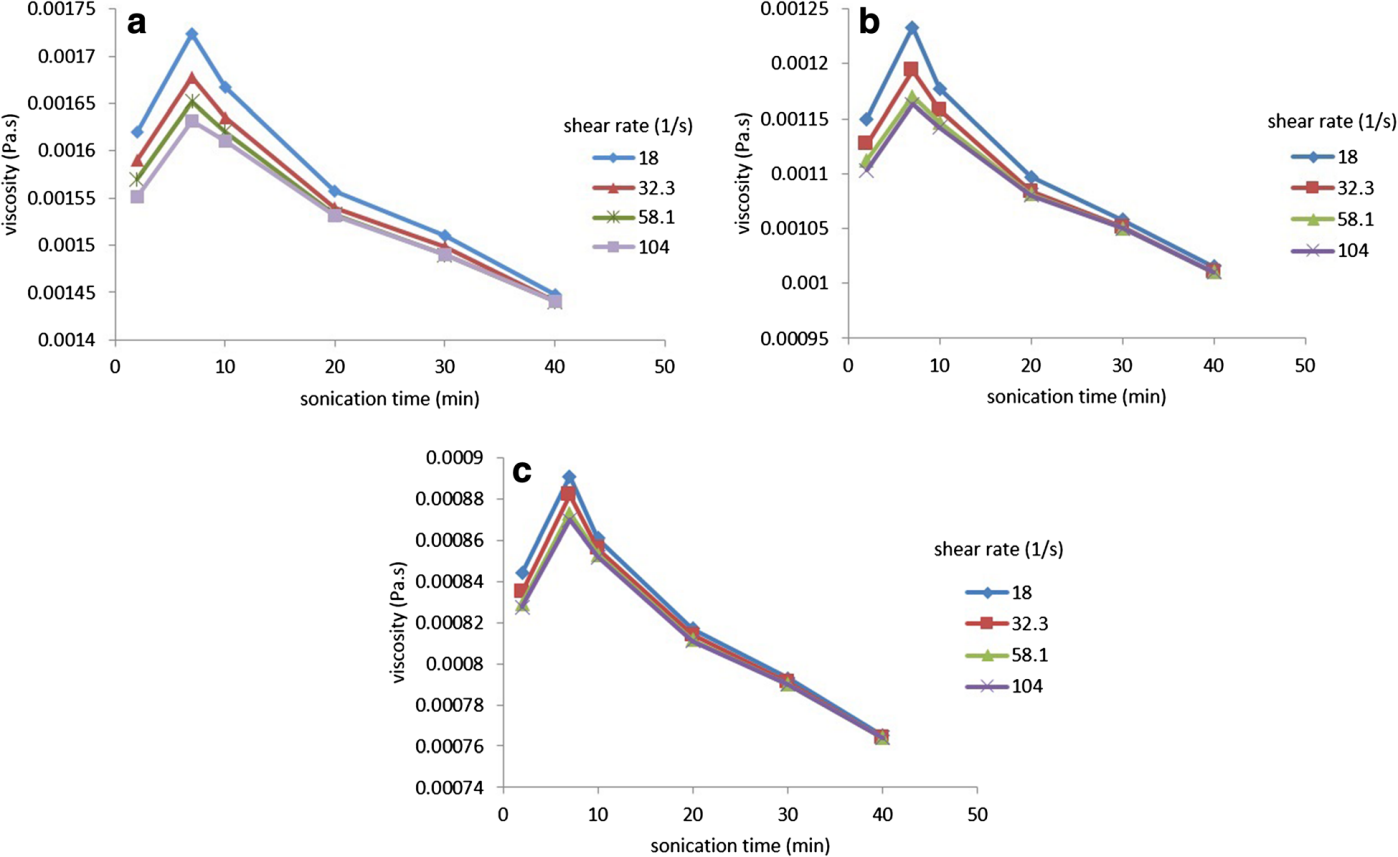
**Variation of dynamic viscosity as function of sonication time at various shear rates. (a)** 15°C. **(b)** 30°C. **(c)** 45°C.

The measured viscosity of MWCNT suspensions is plotted versus ultrasonication time for different shear rates in Figure 13a,b,c. It is seen that the viscosity of the MWCNT nanofluid increases from a lower value for sonication time of 2 min and reaches a maximum value at 7 min sonication time. The viscosity then decreases with further increase in sonication time. As seen from Figure 12, the viscosity of MWCNT nanofluids is higher at low shear rates and decreases with increase in shear rates, which was attributed to the breakup of CNT agglomerates and clusters at high shear rates.

As will be seen in Section ‘Morphology’, the sonication has two important effects. First, it breaks up the clusters and agglomerates and disperses the MWCNTs, which occurs at sonication of about 7 min or longer. Second, at higher sonication times (20 to 40 min), not only the CNT clusters are broken apart but also the MWCNTs are fragmented into smaller pieces. The breakup of the agglomerates into dispersed CNTs and shortening of the length of the CNTs significantly affect the viscosity of the nanofluids.

Well-dispersed CNT nanofluids display high viscosity due to an increase in the surface area of suspended nanoparticles compared to agglomerated and clustered CNT [43]. The presented experimental data shows that a short sonication time (2 min) does not effectively breakup the CNT agglomerates and clusters and the viscosity of the nanofluids is moderately low. A sonication time of about 7 min leads to effective breakup of clusters and dispersion of CNTs in the nanofluid. Figure 13 shows that the measured viscosity for sonication time of 7 min becomes high (compared to a sonication time of 2 min) indicating well-dispersed CNTs in the nanofluid. This figure shows that further ultrasonication (above 7 min) results in a sharp decrease in the viscosity of nanofluids with increasing sonication time. This is because in addition to the breakup of clusters and agglomerates, longer sonication leads to the fragmentation of MWCNTs themselves [7,10,31]. This conjecture is also supported by the observation of TEM images described in Section ‘Morphology’.

**Figure 14:**
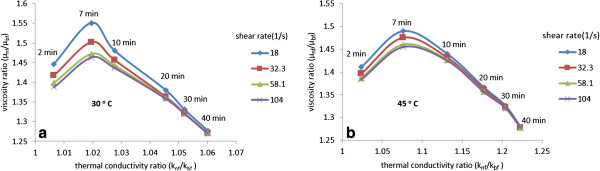
**Variation of viscosity ratio as function of thermal conductivity ratio for MWCNT nanofluid suspensions. (a)** 30°C. **(b)** 45°C.

**Figure 15:**
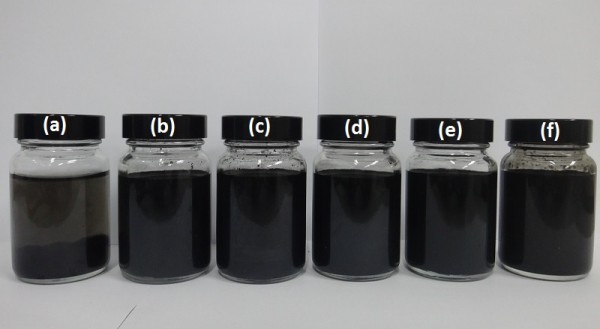
**Digital images of aqueous suspensions of 0.5 wt.% MWCNT dispersed using GA sonicated for different times. (a)** 2 min. **(b)** 7 min. **(c)** 10 min. **(d)** 20 min. **(e)** 30 min. **(f)** 40 min.

The viscosity ratio of MWCNT-water nanofluids as a function of thermal conductivity ratio for different shear rates of 18, 32.3, 58.1 and 104 (1/s) is shown in Figure 14a,b, respectively, for temperatures of 30°C and 45°C. It is seen that the viscosity ratio increases at lower sonication times to a maximum and then decreases sharply with further increase of thermal conductivity ratio and sonication time. Figure 14 shows that the lowest viscosity and highest thermal conductivity of MWCNT-water nanofluids are attained by the extended ultrasonication times of about 40 min. This finding is particularly useful for implementation of nanofluids in practical heat transfer applications, where high conductivity and low viscosity are needed. Digital images of MWCNT suspensions dispersed using GA after 28 days are shown in Figure 15.

### Morphology

**Figure 16:**
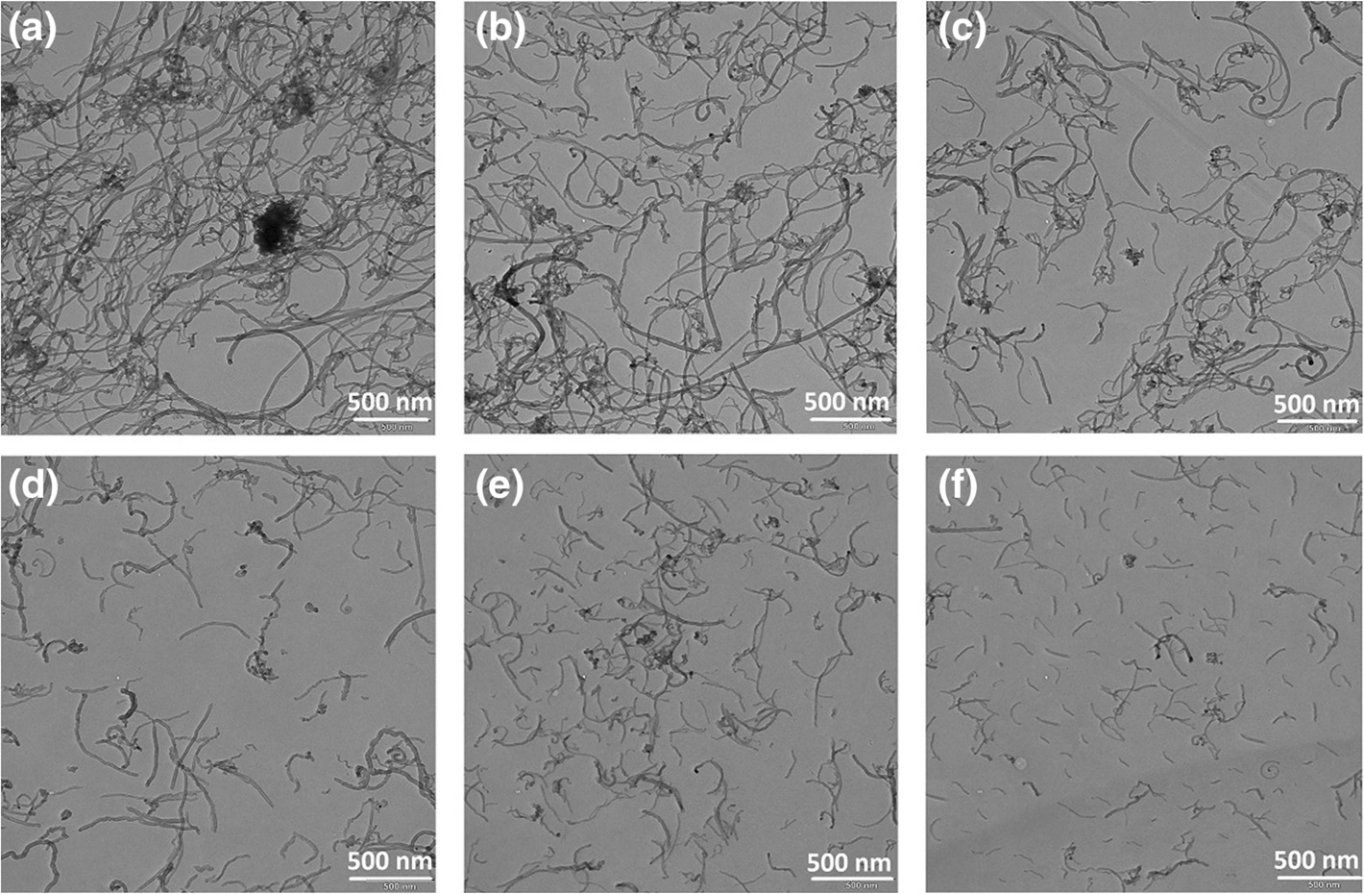
**TEM images of samples (0.5 wt.% MWCNTs, 0.25 wt.% GA) at various sonication times. (a)** 2 min. **(b)** 7 min. **(c)** 10 min. **(d)** 20 min. **(e)** 30 min. **(f)** 40 min.

Figure 16a,b,c,d,e,f displays TEM images of the MWCNT nanofluid samples ultrasonicated, respectively, for 2, 7, 10, 20, 30 and 40 min.

From Figure 16a,b,c, it is seen that there are a number of CNT clusters. In particular, there are a large number of aggregates when the nanofluids were first prepared. The number of clusters is still quite high for sonication time of 2 min and decreases markedly for sonication time of 7 min or higher. Figure 16d,e,f shows that for a high sonication time, there is no agglomerate and the nanotubes are rather uniformly scattered without any noticeable cluster structures. Another important observation from Figure 16 is that as sonication time increases, the mean length of MWCNT in the nanofluid decreases sharply. In particular, Figure 16d,e,f shows that the CNTs are broken into fragments for sonication times of 30 and 40 min. This observation is consistent with the earlier findings of Pohl et al. [29], where an expression for the length of the CNTs as a function of the sonication specific energy, *E*_v_ (sonication energy per unit volume) was suggested, that is,

(1) $$L=AE_{v}^{m}$$

where *L* represents the length of the CNTs, A and m are constants. Yang et al. [32] recommended a modified version of Equation 1 in the case that the sonication specific energy and the volume of nanofluid dispersion are fixed. Accordingly,

(2) $$L=Bt^{n}$$

where B and n are constants and *t* represents the sonication time. The value of n was found to be −0.2742 by Yang et al. [32], which indicates a sharp decrease in the mean length of CNTs with the increase in sonication time [10].

As was noted before, the breakup of clusters by sonication of nanofluids and fragmentation (shortening of mean length) of MWCNT for long duration of sonication significantly affect the effective viscosity and thermal conductivity of the MWCNT nanofluids. In particular, the viscosity at first increases as sonication time increases because of the breakup of clusters and agglomerates and then decreases with further increase of sonication time due the reduction of the mean length of the CNTs.

## Conclusions

The effects of ultrasonication time and type of dispersants on the thermo-physical properties (thermal conductivity and viscosity) of MWCNT nanofluids were investigated in this study. The MWCNT nanofluid suspension containing GA dispersant showed a higher thermal conductivity enhancement compared to suspensions containing SDS and SDBS, which indicated that GA is a proper choice for dispersing MWCNTs. Unlike water, the thermal conductivity of nanofluids initially increased slightly with temperature, followed by a nonlinear increase after 30°C due to increased Brownian motion. The maximum thermal conductivity enhancement was found to be 22.31% at a sonication time of 40 min at 45°C. In general, the thermal conductivity of nanofluids increased with increasing sonication time and reached to its maximum at 40 min, which was the maximum duration studied. This observation was attributed to the breakup of CNT aggregates into smaller dimensions and eventually into fully dispersed MWCNTs.

It was also found that low viscosity and high thermal conductivity nanofluids can be obtained by subjecting the MWCNT-water nanofluids to an extended sonication time of about 40 min. This finding is particularly useful in the implementation of nanofluids in practical heat transfer applications.

It was shown that the MWCNT aqueous solutions exhibited a non-Newtonian shear-thinning behaviour due to the breakup of CNT clusters and agglomerates with increasing shear rate. The viscosity of the nanofluid increased with increasing sonication time up to a maximum value and then decreased with further increase in sonication time. The potential mechanism for the variation of viscosity with sonication time was also discussed. The viscosity of MWCNT suspensions was also shown to decrease with an increase in temperature.

The TEM images revealed the presence of large aggregates in the nanofluids during preparation and even after short time sonication, and the size of these aggregates, however, decreased with increasing sonication time. It was also found that increasing the sonication time reduced the mean length of CNTs and consequently their aspect ratios.

## Competing interests

The authors declare that they have no competing interests.

## Authors' contributions

The manuscript was written through the contributions of all authors RS, GA, HT, MBD, SNK, ES and NZ. All authors read and approved the final manuscript.
